# Differences in the biochemical content and radical scavenging activity of propolis from different parts of a *Meliponula ferruginea* hive

**DOI:** 10.1098/rsos.230241

**Published:** 2023-12-13

**Authors:** Timothy M. Kegode, Hosea O. Mokaya, Solomon I. Chogo, Pierre Noiset, Nicolas J. Vereecken, Amanuel Tamiru, Sevgan Subramanian, Nkoba Kiatoko

**Affiliations:** ^1^ International Centre of Insect Physiology and Ecology (icipe), P.O. Box 30772-00100, Nairobi, Kenya; ^2^ Agroecology Laboratory, Université libre de Bruxelles (ULB), Boulevard du Triomphe CP 264/02, 1050 Brussels, Belgium

**Keywords:** involucrum, sealant, pillars, antioxidant, intra-hive

## Abstract

Research on stingless bee products has increased in recent years, and of particular interest is propolis because of its biological activities such as antioxidant and antimicrobial. However, there is paucity of information regarding intra-hive variations in the biochemical composition and biofunctional properties of this propolis. In this study, we investigated the phytochemicals and radical scavenging activity (RSA) of *Meliponula ferruginea* propolis from 10 wooden hives (*n* = 49). The samples were collected from five different locations comprising the entrance, involucrum, pillars, pots and sealant. Principal component analysis showed that there is an intra-hive variation in phytochemical content and RSA. Phenolic content constituted the highest phytochemical content in all the locations. The sealant and entrance had the highest amounts of phytochemicals compared to the involucrum, pillars and pots. Further analysis of propolis extracts by gas chromatography-mass spectrometry revealed occurrence of different compounds such as monoterpenoids, hydrocarbons, triterpenoids and alkaloids. Hydrocarbons were common in all parts while monoterpenes and triterpenes were present in the entrance. The findings of our study indicates that there is an intra-hive variation in propolis of *M. ferruginea* and hence this information will provide further insight into better understanding of stingless bee propolis.

## 1. Introduction

Propolis is a resinous and complex phytocompound collected by bees from living plants, to serve various physical and physiological functions in a beehive including construction and adaptation of their nests [1]**.** Feral honeybees inhabiting tree cavities also use propolis to smooth the inside of the cavity [2]. It also plays a crucial role in chemical defence against microorganisms [3] and in embalming large dead intruders such as insects and other organisms that are too large to be removed by the bees [4]. The beneficial therapeutic effects of propolis have been recognized and applied since ancient times, with historical records suggesting that its use dates back to ancient Greeks and Romans [5]. The bioactive properties of propolis, such as anti-inflammatory, anticancer, antioxidant, antimicrobial and immune-stimulating activities, have all been demonstrated in previous studies [2]. These biological properties are attributed to the bioactive compounds present in propolis and thought to be associated with the plant secondary metabolites which includes phenolics and terpenoids [1,6]. These bioactive properties play an important role in bee colonies and can be extrapolated to human health benefits.

Stingless bees also produce propolis which has a different name, cerumen [7] and it is suggested that head gland secretions are added to it [8]. Cerumen is plant resin mixed with wax [9], and can also be mixed with soil, resulting in geopropolis [10]. The terms cerumen and propolis are used interchangeably in literature on stingless bees [11]. The most salient characteristic of stingless bees, in contrast to honeybees, is that they use resin as building material for nest elements; pillars, honeypots, and other structures in the nest [12]. They also repel predators and kill would-be invaders [13]. Resin deposited in the vicinity of the colony's nest entrance, entangles termites and ants and thus, prevents invasions [14].

The stingless bee *Meliponula ferruginea* belongs to subgenus *Axestotrigona* with the species being first described by Lepeletier in 1836 and recently by Michener in 2000 [15]. They are widely found in the east African region including Kenya, Tanzania and Uganda [16] and are known to nest in wooden materials and soil. They have no defensive behaviour but notably seal their entrance tube to prevent intruders thus making them easy to handle [15]. In contribution to their conservation in Kenya, they are reared in a simple box hive with a single cavity [17]. The basic nest of *M*. *ferruginea* has the following parts: entrance tube, nest cavity, batumen and involucrum layer, storage pots, brood combs and drainage tube [16]. Batumen resembles a wall and is built by many species to separate the inner part of the nest from the outside environment, it constitutes resins, saps and gums [13], while involucrum, is made of a soft layer of propolis [18]. All these parts have resins incorporated in them but with no clear information on the extent or the choice incorporated at each part. Also, for human health, the potential therapeutic properties such as radical scavenging activity (RSA) of these various parts might vary and information on these intra-hive variations will guide on the parts with the best bioactivities.

The chemical composition of propolis depends on the botanical sources, the availability of plant resins to bees and the bees' preferences [19]. Some studies on *M. ferruginea* propolis indicate that they do not have preference to the kind of resins they use for nest construction [20] and hence, an assumption that all resins in the different hive locations are the same. However, there is no information comparing the biochemical properties of resin from various parts of the nest though a single nest may contain multiple types of resin-rich materials and resin-based structures, each serving a different purpose [13]. Moreover, there is a paucity of information on the biochemical and bioactive properties of *M. ferruginea* propolis. This study investigated the phytochemical composition and RSA of propolis from different locations in the hive. The findings of our study will help to further understand stingless bee propolis and add more knowledge to the scarce information regarding African stingless bees.

## 2. Material and methods

### 2.1. Chemicals

#### 2.1.1. Total phenols

Folin-Ciocalteu's reagent, gallic acid, sodium carbonate (Na_2_CO_3_), ethanol.

#### 2.1.2. Total flavonoids

Quercetin, aluminium chloride (AlCl_3_), sodium nitrite (NaNO_2_), sodium hydroxide (NaOH).

#### 2.1.3. Total terpenoids

Linalool, chloroform, sulfuric acid (H_2_SO_4_), methanol.

#### 2.1.4. Total alkaloids

Colchicine, ferric (III) chloride (FeCl_3_), 1, 10-phenanthroline hydrochloric acid (HCl) and liquid nitrogen.

#### 2.1.5. 2, 2-diphenyl-1-picrylhydrazyl radical scavenging activity

Ethanol, 2, 2-diphenyl-1-picrylhydrazyl (DPPH).

#### 2.1.6. Gas chromatography-mass spectrometry analysis

Dichloromethane.

All purchased from Sigma-Aldrich (Kobian Kenya Ltd.) and were of analytical grade.

### 2.2. Propolis samples

**Figure 1.**
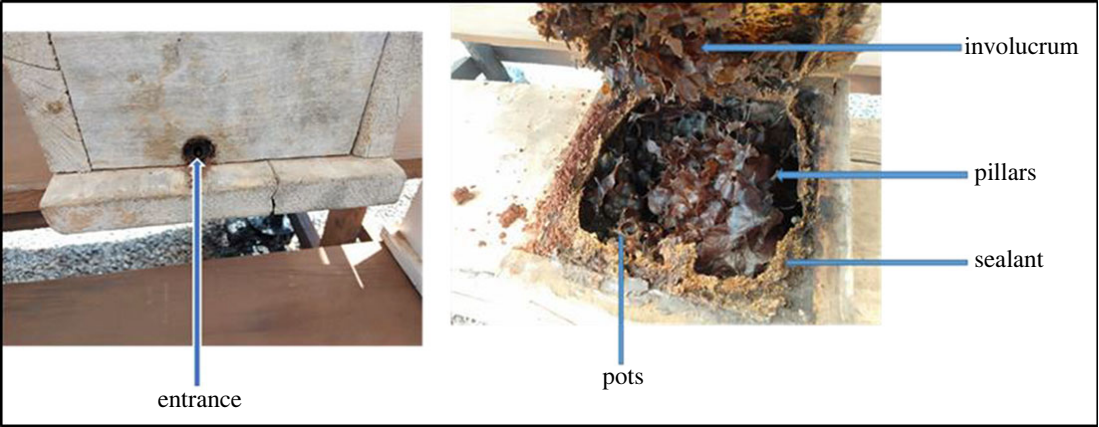
The different locations where the propolis was sampled in the hive. Photo by T. Kegode.

Propolis samples (*n* = 49) were collected directly from 10 wooden *M. ferruginea* hives at *icipe-* Nairobi Duduville campus meliponary (01°13′25.6″ S, 036°53′49.1″ E, 1616 meters above sea level). The hives were opened using a hive tool and the sealant and entrance were scraped off and wrapped in aluminium foil. The other parts, namely, involucrum, pillars and pots were gently cut with sterile scalpel and were wrapped in aluminium. All these parts are indicated in figure 1 [12,13] and tables 1 and 2. The samples were stored at −80°C awaiting chemical analysis.

**Table 1.** Number of samples from each hive part in the 10 hives.

part of hive	entrance	involucrum	pillars	pots	sealant
number of propolis samples (n)	9	10	10	10	10

**Table 2.** Comparison of total content in propolis extracts (means ± s.d.) of the four phytochemicals from the hive locations. (Different superscript letters show significant difference in the content for the different locations. QE, quercetin equivalent; GAE, gallic acid equivalent; LE, linalool equivalent; CE, colchicine equivalent.)

sample	alkaloids content (mg CE/100 g)	flavanoids content (mg QE/100 g)	phenols content (mg GAE/100 g)	terpenoids content (mg LE/100 g)
**involucrum**	640.09 ± 684.16^a^	942.47 ± 190.42^a^	2006.67 ± 252.40^b^	480.44 ± 124.18^ab^
**entrance**	5845.87 ± 293.87^c^	2875.21 ± 166.67^b^	6981.67 ± 415.47^d^	880.67 ± 134.75^d^
**pillars**	368.27 ± 235.43^a^	830.41 ± 292.90^a^	1390.00 ± 237.92^a^	594.00 ± 85.14b^c^
**pots**	2297.11 ± 871.67^b^	906.03 ± 183.28^a^	1593.33 ± 197.01^ab^	453.78 ± 45.80^a^
**sealant**	5398.07 ± 629.56^c^	3046.85 ± 708.60^b^	5376.67 ± 594.72^c^	707.33 ± 134.56^c^
***p*-value**	<0.0001	<0.0001	<0.0001	<0.0001

### 2.3. Sample preparation

Each propolis sample was powdered in liquid nitrogen using a mortar and pestle. The extracts for phytochemical analysis and RSA were then prepared by weighing 0.5 g of each crushed sample in 10 ml of a 50% mixture of ethanol and water (v/v) in a 15 ml Falcon tube and left to soak at room temperature for 72 h. They were vortexed at 3000 rpm for 3 min after every 24 h. After the extraction, they were centrifuged at 600 rpm for 2 min and the supernatant decanted and stored at −80°C.

For gas chromatography-mass spectrometry (GC-MS) analysis, the extraction was done using dichloromethane by dissolving 100 mg of the propolis samples from the different locations of three hives in 10 ml of dichloromethane. The resulting mixture was vortexed at 3000 rpm for 2 min and then incubated for 24 h at room temperature. They were then filtered through glass wool and the filtrate further diluted with dichloromethane by taking 0.1 ml of it in 1.9 ml of dichloromethane.

### 2.4. Quantification of phytochemicals

#### 2.4.1. Total flavonoid content

For the quantification of total flavonoid content (TFC), an aluminium chloride (AlCl_3_) colorimetric assay was carried out as described [21]. In brief, 4 ml of distilled water was added to 1 ml extract of each propolis extract sample, followed by 0.3 ml of 5% (w/v) NaNO_2_ in a test tube. After 5 min, 0.3 ml of 10% AlCl_3_ was added to the mixture and left for another 1 min before adding 2 ml of 1 M NaOH and finally 2.4 ml of distilled water was topped-up. The absorbance was measured using a spectrophotometer (Jenway, Bibby Scientific, Staffordshire, UK) at 510 nm against a reagent blank with water instead of the sample and quercetin was used to generate a calibration curve (20–200 µg ml^−1^), $y\;=\;0.0006x\;+\;0.0028,R^{2}\;=\;0.9981$. TFC was expressed as mg quercetin equivalent per 100 g of propolis (mg QE/100 g propolis). Each sample was assayed in triplicate.

#### 2.4.2. Total phenol content

The Folin–Ciocalteu method was used to quantify total phenol content (TPC) as described [21]. Briefly, 5 ml of 0.2 N Folin–Ciocalteu reagent was added to 1 ml of propolis extract and kept for 5 min before adding 4 ml of 75 g l^−1^ Na_2_CO_3_. The mixture was incubated at room temperature for 2 h and the absorbance of the reaction mixture read at 760 nm against a water blank. This was done in triplicate for each sample. Gallic acid was used as a standard to yield the calibration curve (0–250 µg ml^−1^), $\;y=0.0073x\;+0.0233,R^{2}=0.9992$. The TPC was expressed as mg of Gallic acid equivalents (mg GAE/100 g propolis).

#### 2.4.3. Quantification of alkaloids

The total alkaloid content (TAC) of the propolis extracts was determined using the 1, 10-phenanthroline method as described previously [22]. In brief, 1 ml of 0.025 M FeCl_3_ in 0.5 M HCl and 1 ml of 1, 10-phenanthroline (0.05 M) prepared in 50% ethanol and water mixture (v/v) was added to 1 ml propolis extract, and the mixture incubated for 30 min in a hot water bath at 70°C. The absorbance of red coloured complex was measured at 510 nm against reagent blank. This was done in triplicate for each sample. Alkaloid contents were determined using a standard curve of colchicines (0.1–1.5 mg ml^−1^), prepared by dissolving 20 mg of colchicine in 10 ml of a 50% mixture of ethanol and water (v/v) and diluted to the required concentration, $y\;=\;1.866x\;+\;0.2332,R^{2}\;=\;0.9844$. The results were expressed as mg of colchicine per 100 g of propolis (mg CE/100 g propolis).

#### 2.4.4. Quantification of terpenoids

The total terpenoid content (TTC) was quantified using the colorimetric method [23]. This was carried out by adding 1.5 ml of chloroform to 200 µl of the propolis extract and the mixture vortexed at 3000 rpm for 3 min. Thereafter, the mixture was incubated in the dark for 90 min without disturbance after adding 100 µl of concentrated H_2_SO_4_. The supernatant was decanted gently to leave a reddish brown precipitate at the bottom and 1.5 ml of absolute methanol added and vortexed again until the precipitate dissolved completely. Each sample was assayed in triplicate. The same procedure was repeated for the standard using different concentrations of linalool (10 mg ml^−1^–500 mg ml^−1^), $\;y\;=\;0.0009x\;-\;0.0158,\;R^{2}\;=\;0.9914$ and the absorbance measured at 538 nm with methanol as a blank. The TTC was calculated using linalool solution as the calibration curve and expressed as linalool equivalents in 100 g propolis (mg LE/100 g propolis).

### 2.5. Analysis of *in vitro* 2, 2-diphenyl-1-picrylhydrazyl radical scavenging activity

The DPPH assay was carried out as described [24]. Briefly, 3 ml of DPPH solution (2 mg/100 ml ethanol) was added to 1.5 ml of propolis extract obtained by diluting the initially prepared extract in the ratio of 1 : 199. The mixture was then incubated for 15 min at 37°C in the dark and the absorbance measured at 517 nm against a control of 1.5 ml of ethanol mixed with 3 ml DPPH solution. Each sample was assayed in triplicate. A quercetin standard was prepared (10–100 µg ml^−1^), $y\;=\;0.591x\;+\;38.413,\;R^{2}\;=\;0.9988\;$ (electronic supplementary material, table S3 and figure S1). The free RSA was expressed as a percentage of inhibition, using the following formula:

$$\text{\%}\mathrm{radical}\;\mathrm{scavenging}\;\mathrm{activity}=\left[\frac{\mathrm{control}\;\mathrm{absorbance}\text{–}\mathrm{sample}\;\mathrm{absorbance}}{\mathrm{control}\;\mathrm{absorbance}}\right]\times 100\text{\%}.$$

### 2.6. Gas chromatography-mass spectrometry analysis of propolis extracts

GC-MS analysis of propolis extracts was carried out using a GC (HP-7890A, Agilent technologies, USA) directly coupled with spectrometer (MSD-5975C, Agilent technologies, USA). Chromatographic separations was achieved by an HP5-MSI capillary column, 30 m in length × 0.25 mm in diameter, 0.25 µm film thickness (J & W Scientific, Folsom, USA) immobilized with 5% phenyl methyl silicone as the stationary phase. An aliquot (2 µl) of diluted propolis sample was injected into the GC-MS instrument, in the splitless mode, using an auto sampler (7683B series, Agilent technologies, USA) at an injector temperature of 270°C.The sample was then transported by Helium gas (99.99% purity) as the carrier gas at a flow rate of 1.2 ml min^−1^. The oven temperature was programmed at 35°C where it was held for 5 min followed by a gradual increase at the rate of 10°C min^−1^ to 280°C where it was held at an isothermal state for 10 min. The transfer line temperature was at 280°C while the temperature of the ion source was 230°C and the ionization energy was 70 eV. Fragment ions analysed in scan mode over 40–450 *m/z* mass range and MS quadrupole temperature was maintained at 150°C.

### 2.7. Data analysis

The R working environment v 3.5.0 (R Core Team 2019) along with packages *factoextra* 1.0.5 and *ggplot2* 3.1.1 [25,26] was used to perform a principal components analysis (PCA) and visualize the phytochemical composition of propolis from the different hive location. To compare the phytochemical contents we ran a Kruskal-Wallis test followed by *post-hoc* Dunn's test after confirming that the data for the parameters were not normally distributed. A table of means and boxplots were also prepared using package *ggplot2* 3.1.1.

The relative integration of each identified peak was determined using the ChemStation integrator. To eliminate potential column, or solvent contamination, blank runs were conducted and subsequently analysed. Detected peaks were initially identified through a comparative analysis of mass spectral data against reference spectra published by library–MS databases: National Institute of Standards and Technology (NIST) available versions of 2005, 2008 and 2011 as well as by considering retention times. When authentic standards were available, the compound identifications were definitively confirmed by comparing their fragmentation patterns and retention times, with those of commercially available standards.

The data presented had a similarity structure compound estimate (similarity index) ≥90% (table 3). Prediction of biological activity of the compounds was based on Dr Duke's Phytochemical and Ethnobotanical Databases [27].

**Table 3.** Compounds present in propolis from different parts of the hive identified by GC-MS analysis. (Only compounds with similarity score ≥ 90% were considered. The table shows compounds present in in propolis and their different classes. (+) compound present, (−) compound absent.)

compound	retention time (min)	compound classification	biological activity	entrance	sealant	pillars	cerumen	pots
1,2-benzisothiazol-3-amine	50.53	alkaloid	antioxidant	−	+	−	−	−
octadecahydro-2H-picen-3-one	37.36	triterpenoid	antioxidant	+	+	+	+	+
octadecane^a^	33.34	hydrocarbon	antioxidant	+	+	+	+	+
nonadecane^a^	34.83	hydrocarbon	antioxidant	+	−	+	+	+
hexadecanoic acid^a^	23.22	fatty acid	antioxidant	+	−	−	−	−
eicosene	30.82	hydrocarbon	antioxidant	−	−	+	−	−
eicosane^a^	29.84	hydrocarbon	antifungal	+	+	+	+	+
pinene^a^	9.23	terpenoids	antioxidant	−	+	−	−	+
heneicosane^a^	24.52	hydrocarbon	antibacterial	+	+	+	+	+
docosane^a^	28.66	hydrocarbon	antioxidant	+			+	−
tricosane^a^	26.26	hydrocarbon	no activity reported	−	+	+	−	+
tetracosane^a^	26.26	hydrocarbon	antioxidant	+	−	+	+	+
pentacosane^a^	27.89	hydrocarbon	antioxidants	−	−	+	+	−
hexacosane^a^	28.66	hydrocarbon	antioxidants	−	−	+	+	+
octacosane^a^	34.47	hydrocarbon	antioxidants	+	+	+	+	+
terpinene^a^	9.23	terpenoids	antioxidant	−	+	−	−	−
octadecenoic acid^a^	25.15	fatty acid	antioxidant	−	−	+	−	−
acetic acid^a^	39.43	carboxylic acid	antibacterial	+	−	−	−	−
antra-9,10-quinone	47.47	alkaloid	antioxidant	−	+	−	−	−
*cis*-11-eicosenoic acid^a^	26.64	fatty acid	anti-inflammatory	+	+	−	−	−
cyclohexane-1,3-dione	48.47	alkaloid	anti-inflammatory	+	+	+	+	+
friedours-7-en-3-one	39.85	triterpenoid	antioxidant	−	+	+	−	−
friedelan-3-one	41.89	triterpenoid	anti-infammatory	+	−	−	−	−
lanosta-8,24-dien-3-one	36.98	triterpenoid	antioxidant	+	+	+	+	+
lanosterol	37.58	triterpenoid	antioxidants	−	−	−	+	+
lup-20(29)-en-3-one	38.39	triterpenoid	antimicrobial	−	−	−	−	+
octadecanoic acid^a^	25.09	fatty acid	antioxidant	+	+	−	−	−
olean-12-ene	41.24	triterpenoid	antioxidant	+	−	−	−	−
oxirane, hexadecyl-	32.59	alkaloid	antioxidant	−	−	+	+	+
phenol, 2,4-bis(1,1-dimethylethyl)-	18.36	phenolic	antioxidant	−	−	+	−	−
pyridine N-oxide, 2-amino-3,5-dibromo-	34.97	alkaloid	antioxidant	−	+	−	−	−
pyridine-3-carboxamide	33.62	alkaloids	antioxidant	+	+	+	+	−
squalene^a^	30.46	triterpenoid	antioxidant	−	+	−	−	−
supraene^a^	30.46	triterpenoid	antioxidant	−	−	−	−	+
taraxasterol	40.91	triterpenoid	antioxidant	−	−	+	−	−
ursa-9(11),12-dien-3-one	35.68	triterpenoid	antibacterial	+	+	−	−	−

^a^Compounds identified through authentic standard.

## 3. Results

### 3.1. Quantification of phytochemicals

The quantified total phytochemicals content based on the colorimetric assays showed significant variation in all the parts inside the hive, *p* < 0.0001 (table 2). Based on the individual hive parts, the sealant and entrance had the highest quantity of the phytochemicals compared to pillars, involucrum and pots. In general, phenolic content was the highest among the individual phytochemicals analysed in propolis extracts followed by alkaloids, flavanoids and terpenoids. The mean total quantity of flavanoid content was between 830.41 and 3046.85 mg QE/100 g, phenol content from 1390.00 and 6981.67 mg GAE/100 g, alkaloid content from 368.27 and 5398.07 CE mg/100 g, and terpenoid content from 453.78 to 880.67 mg LE /100g.

**Figure 2.**
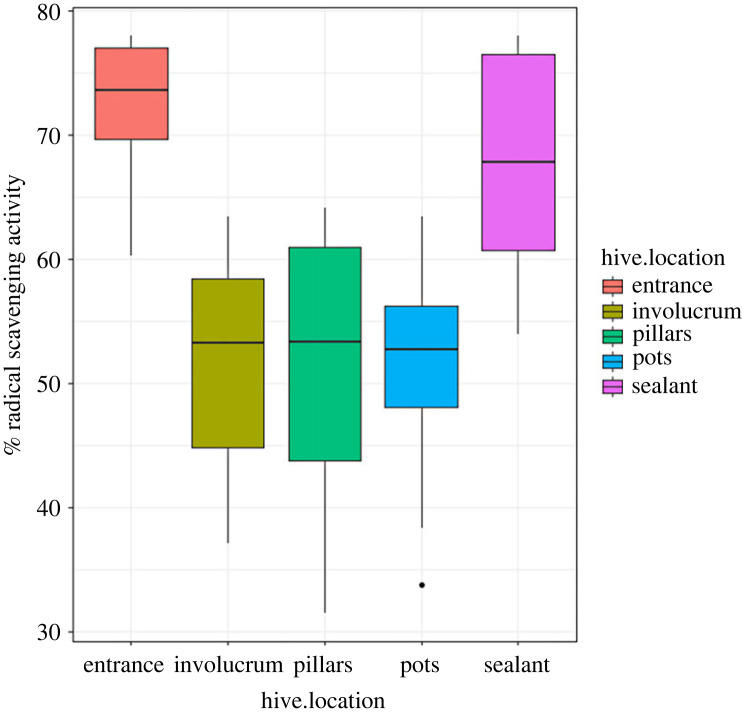
Principal component analysis on the phytochemicals and the radical scavenging activity of propolis samples from the different hive locations.

From the PCA plot (figure 2), there is a separation of propolis phytochemicals in respect to the different parts of the hive. The major separation is along the first dimension (dim. 1) that explains 85.3% of the variation and separates the nest locations into two, with sealant and entrance on the positive side while pillars, pots and involucrum are on the negative side. The parts on the positive side are on the outside of the hive while the others are on the inner side. The minor separation is along the second dimension (dim. 2) that explains 10.3% of the variation and separates the components based on phytochemical content with terpenoids on the positive side and flavonoids, alkaloids and phenols on the negative side. The RSA in the PCA is in closer to flavonoids and phenols indicating that these phytochemicals are more responsible for the activity.

#### 3.1.1. Radical scavenging activity

**Figure 3.**
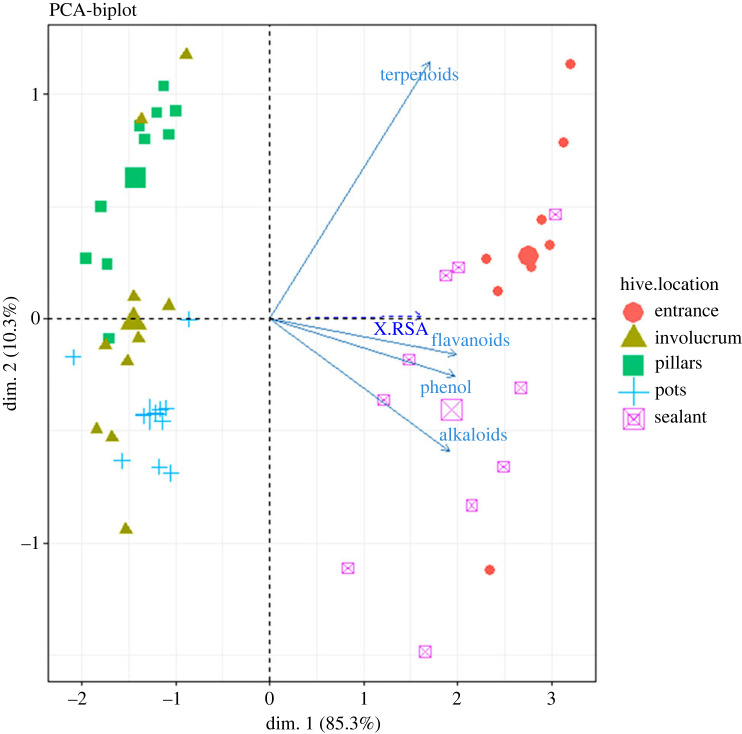
Radical scavenging in percentage of the different propolis from the different locations in the hive. The horizontal lines represent the mean while the upper and the lower vertical lines represent the range of the radical scavenging activity.

Radical scavenging activity (% RSA) is the percentage of DPPH radical molecules that has been reduced to stability rendering it non-reactive. From the box plots (figure 3), there is a difference in the ability of the propolis extracts to reduce DPPH ranging from 30 to 80% RSA for 250 mg l^−1^ of propolis extract. The observed variation is location specific with the sealant, and entrance having all samples with high antioxidant activity compared to pillars, pots and involucrum. The quercetin standard equivalent for the propolis extracts had the highest value of 67.30 µg ml^−1^ (electronic supplementary material, table S2). This is in accordance with the phytochemical data where the same have high total content of the phytochemicals.

#### 3.1.2. Gas chromatography-mass spectrometry analysis

**Figure 4.**
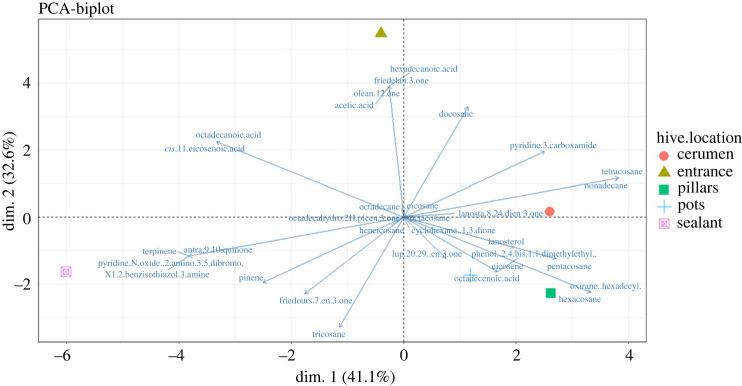
Principal component analysis on the GC-MS compounds of propolis extracts from the different hive locations.

Analysis of GC-MS chromatograms of the five hives with samples representing each location showed more than a hundred peaks indicating the presence of various compounds (electronic supplementary material, table S3). These compounds belong to different classes such as hydrocarbons, terpenoids, alkaloids, triterpenoids, fatty acid and phenols (table 3). The number of detected compounds varied in each class. Compounds classified as terpenoids were the most abundant followed by hydrocarbons, alkaloids and phenols classes of compounds were the least. Chemical analysis revealed clear difference in the chemical composition of propolis present in the various parts of the hive, while those in the sealant and entrance exhibiting higher compound diversity compared to the other parts. This variation was further confirmed by our binary data PCA plot (figure 4), which explained 73.7%. The major separation is along dim. 1 that explains 41.1% of the variation and separates the nest locations into two with compounds classified as fatty acids being on the negative side except for octadecenoic acid. Also, terpenes were separated on the negative side. Contrary, hydrocarbons were separated to the positive side. The minor separation is along dim. 2 that explains 32.6% of the variation, separating again the hive location into two with terpenes such as pinene and terpinene and hydrocarbons being on the negative side while the fatty acid compounds are on the positive side. Based on the hive locations, there is a distinct separation in accordance to the chemical classes present. The entrance is observed to be associated with fatty acid compounds while the sealant is associated with the terpenes. The hydrocarbons are present in all the hive locations but closely associated with the cerumen, pillars and pots. Most of these compounds have been reported to possess radical scavenging activities (table 3).

## 4. Discussion

### 4.1. Quantification of phytochemicals

Varying amounts of phytochemicals illustrate the complex chemical composition of propolis as a mixture of plant resins and bee secretions [28]. In stingless bees, it has been reported that they also add head secretions to their propolis [8] which further enriches its chemical composition. These bees have a vestigial sting making them vulnerable, but in turn they have developed adaptations which entails the use of chemicals that are self-produced or externally acquired [29]. Among the adaptations, resins incorporated into the propolis serves as source of external chemicals and its diversity helps in serving the different functions [30]. The major uses of resins are structural and defence, in which they place resin droplets at the nest entrance [31]. Noticeably, it has been observed that freshly collected resin appears to play a critical role in nest defence against intruders such as ants [32]. The composition and concentration of phytochemicals is dependent on many factors such as bee species, and bees preference to certain plants, which is influenced by the local climatic conditions [33]. These compounds are secondary metabolites derived from plants, and are produced to serve various roles. We observed that the location of propolis in the hive has influence on the total content of the phytocompounds although many studies have not always indicated the location in the hive where the stingless bee propolis had been sampled. When we consider the entrance and sealant, the total phenol and flavonoid content agrees with the values reported for Malaysian *Geniotrigona thoracica* propolis of 29.1 mg g^−1^ GAE and 19.78 mg g^−1^ QE [34]. The phytochemical concentrations in pillars, pots, and cerumen were lower than the listed values reported by other researchers. Stingless bee honey from the same region has TFC of 28.7 to 73.0 mg QE/100 g and TPC of 57.0 to 214 mg GAE/100 g which is lower than our results [35]. In comparison with Indonesian stingless bee propolis, 10 to 28.65 mg g^−1^ GAE and 0.16 to 3.39 mg g^−1^ QE TFC are in the range of our results including pillars, pots and cerumen [36].

Monoterpenes and sesquiterpenes are among the terpenoids that make up the volatile components of the propolis and are known to produce the aroma given off by the propolis [33]. Terpenoids in propolis plays a major role as insect pest repellents explaining why their concentration in the entrance and sealant is higher [29]. It has been reported in some studies that propolis with fresh resins has monoterpenes that is able to repel ants and other vertebrates [32] affirming its use at the entrance of the hive. They also play a major part in the biological activities of the propolis essential oil extract, such as antimicrobial and anti-inflammatory activities [33]. The TTC in Indian propolis has been reported to be 1.5 to 3.8 LE mg/100 g, which is lower compared to our results [37]. The difference with our results can be attributed to the variation in vegetation. Alkaloids were first discovered and isolated from propolis in 2015, and only 16 alkaloids from Brazil and Algeria had been identified to date [38]. Studies on the TAC are limited, with some stating the absence of alkaloids as in the case of Indonesian *Trigona sp*. propolis extracts [39]. Indian *Apis mellifera* propolis extract has values ranging from 6.2 to 9.8 mg/100 g which is lower compared to our results [40]. Alkaloids are secondary metabolites produced and stored in various parts of plants. They play an important role in deterring herbivores which include mammals and insects [41]. These properties can be extrapolated to their function in propolis as a way of chemical defence against insects.

### 4.2. Radical scavenging activity

Tests for RSA such as the DPPH assay has been extensively applied to determine the RSA of extracts and pure substances [42]. Its stability as a free radical compound forms a solution with a deep violet colour [43]. DPPH reacts with any molecule that has the ability to donate an electron or hydrogen to it. This reaction can be monitored by measuring the decrease in absorbance at 517 nm [43]. The antioxidant activities of terpenoids, phenols, and flavonoids are well documented [33]. The concentration of 250 mg l^−1^ in our study promoted varying antioxidant activity, with the highest being 80%. The higher the percentage recorded means the extract has a higher antioxidant activity. Our results are in accordance to those that have been reported in different stingless bee species [44]. Propolis has been reported to be a good source of natural antioxidants as a result of its rich phytochemical composition [43]. Plant antioxidants are synthesized by plants to counteract biotic stress and favour the attraction of pollinators which ends up in the propolis [45]. These antioxidants possess bioactive properties with phenolic being the most abundant with the highest RSA [46]. We adopted in our study to further corroborate our results on phytochemicals.

### 4.3. Gas chromatography-mass spectrometry analysis

In this study, the propolis samples had varying compound profiles as reported in other studies [47]. There was variation in the chemical composition of propolis in accordance to the different locations in the hive. These compounds belonged to different classes of phytochemicals including triterpenoids, hydrocarbons, fatty acids and pyridine derivatives which have also been reported in stingless bee propolis [48]. Some phytochemicals such as octadecahydro-2H-picen-3-one, acetic acid, tricosane have been reported to be present in propolis [49]. Apart from the difference in phytochemical composition, there was variation in the classes of compounds present in each part of the hive. The chemical composition of propolis is directly affected by vegetation [19]. The presence of long-chain hydrocarbons such as eicosane in the propolis have also been reported in other studies [40]. Propolis composition constitutes resins, demonstrating that it contains compounds of plant origin such as alkaloids—which have been reported in studies on plants [41]. The absence of other compounds such as the flavonoids in our results is because we did not carry out derivatization of our samples. This is a limitation as we could only report a limited number of compounds. When we consider these chemical profiles, there is a clear difference, with sealant and the entrance being distinct from the other hive parts.

## 5. Conclusion

The outcome of our study suggests that the biochemical content of *M. ferruginea* propolis exhibits intra-hive variation in accordance to its location in the hive. This is corroborated by the total phytochemical content and GC-MS analysis of the propolis extracts. The difference can be attributed to the various functions of propolis in the hive leading to different resins being incorporated. The different compounds also result in varying RSAs. Our work provides additional information to the existing knowledge on stingless bee propolis since it is, to our knowledge, the first report on stingless bee intra-hive variation in phytochemical composition and RSAs of *M. ferruginea* propolis.

## Data Availability

The datasets supporting this article have been uploaded as part of the electronic supplementary material [50].
